# Detecting the Type and Severity of Mineral Nutrient Deficiency in Rice Plants Based on an Intelligent microRNA Biosensing Platform

**DOI:** 10.3390/s25165189

**Published:** 2025-08-21

**Authors:** Zhongxu Li, Keyvan Asefpour Vakilian

**Affiliations:** 1 College of Information and Electrical Engineering, China Agricultural University, Beijing 100083, China; 2022308250101@cau.edu.cn; 2 Department of Biosystems Engineering, Gorgan University of Agricultural Sciences and Natural Resources, Gorgan 4913815739, Iran

**Keywords:** biosensor, feature selection, microRNA compounds, random forest

## Abstract

The early determination of the type and severity of stresses caused by nutrient deficiency is necessary for taking timely measures and preventing a remarkable yield reduction. This study is an effort to investigate the performance of a machine learning-based model that identifies the type and severity of nitrogen, phosphorus, potassium, and sulfur in rice plants by using the plant microRNA data as model inputs. The concentration of 14 microRNA compounds in plants exposed to nutrient deficiency was measured using an electrochemical biosensor based on the peak currents produced during the probe–target microRNA hybridization. Subsequently, several machine learning models were utilized to predict the type and severity of stress. According to the results, the biosensor used in this work exerted promising analytical performance, including linear range (10^−19^ to 10^−11^ M), limit of detection (3 × 10^−21^ M), and reproducibility during microRNA measurement in total RNA extracted from rice plant samples. Among the microRNAs studied, miRNA167, miRNA162, miRNA169, and miRNA395 exerted the largest contribution in predicting the nutrient deficiency levels based on feature selection methods. Using these four microRNAs as model inputs, the random forest with hyperparameters optimized by the genetic algorithm was capable of detecting the type of nutrient deficiency with an average accuracy, precision, and recall of 0.86, 0.94, and 0.87, respectively, seven days after the application of the nutrient treatment. Within this period, the optimized machine was able to detect the level of deficiency with average MSE and R^2^ of 0.010 and 0.92, respectively. Combining the findings of this study and the results we reported earlier on determining the occurrence of salinity, drought, and heat in rice plants using microRNA biosensors can be useful to develop smart biosensing platforms for efficient plant health monitoring systems.

## 1. Introduction

While environmental stresses significantly influence a wide range of plant morphological, physiological, and biochemical traits, these changes are often not unique to a specific type of stress [1]. For example, different stressors can cause similar effects on plant height as well as root and shoot biomass [2]. To gain a deeper understanding of how plants respond to stress at the molecular and cellular levels, it is important to focus on traits that display more stress-specific patterns. In recent years, microRNA expression has been explored as a tool to study plant responses under various stress conditions [3].

microRNAs are small non-coding RNA molecules, typically consisting of 17 to 23 nucleotides, that play a significant role in regulating gene expression in living organisms [4]. Despite their short length, they target genes involved in critical physiological and biochemical processes in plants, including growth, hormone regulation, signal transduction, and responses to various stresses [5,6]. Common abiotic stress factors affecting plants include light intensity, temperature fluctuations, nutrient imbalances, drought, salinity, and elevated CO_2_ levels [7]. The expression of microRNAs under stress conditions is typically specific to the plant tissue and the severity or stage of the stress [8]. Identifying stress-responsive microRNAs enhances our understanding of their involvement in improving plant tolerance mechanisms [9,10]. Investigating microRNA-regulated gene networks is key to comprehending how plants respond to environmental challenges [11]. Over the past decade, extensive research has focused on how microRNA target gene structures influence stress responses [12]. Some microRNAs are known to regulate responses to multiple stress types, while others appear to respond specifically to a single stress factor or have only one known function identified so far [13].

Rice (*Oryza sativa* L.) is a staple food crop globally and forms the foundation of many rice-based diets. One of the major challenges in rice cultivation is mineral nutrient deficiency, which can severely impact plant physiological and biochemical functions [14]. Nitrogen (N), a crucial macronutrient essential for chlorophyll production and the synthesis of amino acids and proteins, is particularly important. Its deficiency often leads to stunted growth, yellowing or pale green older leaves, reduced tillering (fewer side shoots), poor grain filling, and low yields [15]. Phosphorus (P) deficiency typically results in dark green or purplish foliage, especially in seedlings, along with weak root systems, delayed flowering and maturity, and reduced tillering and yield [16]. Potassium (K), which is vital for water regulation, enzyme activation, and disease resistance, when deficient, causes brown leaf edges, weak stems, vulnerability to pests and diseases, and suboptimal grain filling [17]. Sulfur (S) is also key for protein synthesis, and its shortage can cause yellowing of young leaves, stunted growth, and poor panicle formation [18].

Several microRNAs have been identified in rice plants that are involved in plant responses to the deficiency of these nutrients. Studies have shown that some microRNAs, such as miRNA169 and miRNA444, targeting NF-YA and MADS-box transcription factors, are involved in N deficiency [19]. Moreover, miRNA399 and miRNA827, with target proteins of PHO2 and SPX-MFS, exert changes in expression toward P deficiency [20]. miRNA319, by targeting TCPs, and miRNA444 are involved with K deficiency [21]. miRNA395, targeting ATP sulfurylases and sulfate transporters, regulates S assimilation and transport. Other general stress-responsive microRNAs in rice plants include miRNA156, miRNA159, miRNA162, miRNA164, miRNA167, miRNA171, miRNA172, and miRNA528 [22]. Although the functions of most transcription factors in rice have been well studied, the comprehensive function of all of them in determining the morphological and physiological status of the plant against abiotic stresses such as nutrient deficiency still requires extensive studies [23].

Various techniques, such as microarrays [24], Northern blotting [25], and polymerase chain reaction (PCR) [26], have been employed to analyze microRNA expression in plants. However, these methods are often limited by factors such as low sensitivity, narrow linear detection range, and limited detection thresholds [27,28]. To overcome these challenges, biosensors—devices incorporating living bioreceptors—have emerged as a more reliable and sensitive alternative for microRNA detection [29]. Recently, a variety of electrochemical biosensors with broad linear ranges and high sensitivity have been introduced, capable of detecting microRNA concentrations down to femtomolar levels [30,31].

Machine learning techniques have emerged as a significant advancement in plant sciences, where they are used to model plant behavior across various stages, including planting, storage, harvesting, and postharvest processes [32,33,34]. These methods can effectively learn complex, multivariable relationships between input and output data through training datasets [35]. With the increasing size of data in plant stress studies, machine learning can be considered an essential tool for analyzing the roles of microRNAs in both biotic and abiotic stress responses. Recently, several research efforts have led to the development of software tools specifically designed to predict microRNA functions related to plant adaptation to abiotic stresses [36,37,38].

In our previous work [39], we investigated the possibility of detecting the occurrence of environmental stresses in rice plants by having the concentration of microRNAs measured in plant samples using an optical biosensor. Based on our results, miRNA156, miRNA393, and miRNA159 exerted the greatest contribution in predicting drought, salinity, and heat stress in the studied rice plants, respectively. Moreover, the support vector machine (SVM) was able to predict the level of salinity, drought, and heat with coefficients of determination equal to 0.94, 0.91, and 0.86, respectively. In addition to rice plants, we also tried to detect environmental stress factors, such as temperature, salinity, and drought, in Arabidopsis as a model plant using microRNAs measured by an optical biosensor [40].

However, a literature review shows that no similar work has been carried out on the detection of mineral nutrient deficiency in plants using smart microRNA biosensing platforms. In addition, no work has reported a comprehensive analysis (including linear range, limit of detection, and reproducibility) of electrochemical schemes for the measurement of microRNAs in plant samples. Considering the fact that electrochemical setups are more cost-effective than optical setups in their development as a portable device, this study pursued three main objectives: (a) to investigate the performance of an electrochemical biosensing platform that has always being used for analyzing microRNAs in human samples but now for the measurement of microRNAs in plant samples; (b) to identify the effective microRNA compounds in plants’ response to mineral nutrient deficiencies; and (c) to develop an efficient machine learning scheme for the detection of type and severity of the deficiencies by having the concentration of effective microRNAs measured in plant amples as machine inputs. In this case, based on the results obtained in this work and our previous studies, an electrochemical device can be fabricated for the efficient monitoring of environmental stresses in rice plants.

## 2. Materials and Methods

**Figure 1:**
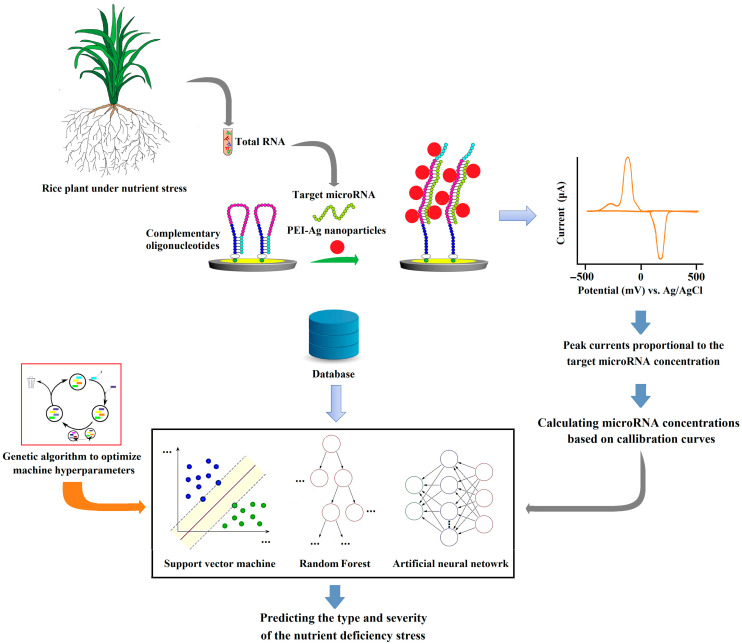
The proposed method for predicting stresses in rice plants by using microRNA concentrations and machine learning.

Figure 1 shows the steps of this work. First, nutrient deficiency at various levels, i.e., mild, moderate, and severe, were applied to young rice plants separately and simultaneously, and the concentrations of various rice microRNAs, which have been identified in previous studies as compounds related to the plant response to such stresses, were measured in the shoot of the plants. Then, after identifying the most effective features using a feature selection scheme, the features were considered input variables of SVM, random forest (RF), and artificial neural networks (ANNs), optimized by a genetic algorithm (GA) to predict the type of stress and its severity.

### 2.1. Plant Materials, Growth Conditions, and Treatments

Plant cultivation was carried out in a research field 10 km from Gorgan, Golestan Province, Iran. Rice seeds (*Oryza sativa* cv. Fajr) were disinfected with sodium hypochlorite (Merck, Darmstadt, Germany) and stored in Murashige and Skoog culture medium for 7 days. After germination, rice seedlings were grown in the complete Yoshida solution for 21 days at 28 °C. The solution was changed every three days. Then, the seedlings were subjected to nutrient deficiency treatments by modifying the concentration of ammonium nitrate, sodium phosphate, potassium sulfate, and magnesium sulfate as the supplies of N, P, K, and S, respectively, in the complete Yoshida solution. To apply mild, moderate, and severe stress levels, the elemental supplies were reduced by 20, 50, and 100% for 8 days, where no change in the supplies was applied for control. Under strong stress conditions, significant leaf curling was observed. Although, in general, only one nutrient should be deficient per treatment to isolate effects, the interaction of various nutrient deficiencies is investigated in this work to further study the role of microRNAs in multiple stress conditions and to assess the capability of machine learning in accurate and reliable detection of the type and severity of the stress. The experiment was performed with five replicates to create a suitable database for training machine learning methods. After sampling the plants, the samples were stored in a freezer at −80 °C to extract the total RNA content.

### 2.2. microRNA Measurement Using the Biosensor

The microRNA concentrations in plant extracts were measured using an electrochemical biosensing platform proposed by Hakimian and Ghourchian [41]. In this technique, target microRNAs hybridize with complementary oligonucleotides immobilized on the electrode surface, leading to an increase in negative charge near the working electrode. This enhanced negativity facilitates the adsorption of positively charged polyethyleneimine–silver (PEI–Ag) nanoparticles, which act as electroactive labels, onto the microRNA/oligonucleotide hybrids. The resulting increase in electric peak current correlates with the concentration of the target microRNA. The concentrations of miRNA156, miRNA159, miRNA162, miRNA164, miRNA167, miRNA169, miRNA171, miRNA172, miRNA319, miRNA395, miRNA399, miRNA444, miRNA528, and miRNA827 were measured in plant samples in this work.

PEI (50% solution, Mn ~ 1200, Mw ~ 1300), silver nitrate, and phosphate-buffered saline (PBS) (0.1 M, pH = 7.5) were purchased from Merck (Darmstadt, Germany). The target microRNAs (with sequences obtained from https://mirbase.org, accessed on 20 November 2024) and their thiolated HPLC-purified probes utilized in the present research were purchased from Zistfanavar Noandishan (Tehran, Iran). The sequences of target microRNA and its thiolated probe are displayed in Table 1.

To synthesize PEI–Ag nanoparticles, 100 mL of 10 mM silver nitrate solution was mixed with 1 mL of 2% (*w*/*w*) PEI and brought to a boil while stirring. After 15 min, the solution turned green. It was then centrifuged at 8000× *g* for 5 min, and the sediment was washed using an ultrasonic homogenizer with deionized water for 1 min [42]. Thiolated probes were immobilized on the working electrode via their thiol groups. For this, 8 μL of a 1 μM probe solution was mixed with 8 μL of PBS, applied to the electrode, dried under infrared light for 30 min, and then rinsed three times with deionized water.

To begin the hybridization process, the biosensor electrode was immersed in a 0.1 M PBS solution at 60 °C for 30 s. Immediately after that, 20 μL of the sample containing the target microRNA was mixed with 20 μL of PBS and applied to the electrode surface, allowing the target microRNAs to hybridize with the immobilized probes. The electrode was then dried under an infrared lamp and rinsed with deionized water. Next, 1.8 μg of positively charged PEI–Ag nanoparticles (in 6 μL of deionized water) was added to the electrode surface. These nanoparticles bound to the negatively charged probe–target microRNA hybrids. After another drying and washing step, cyclic voltammetry was performed in 2 mL of 0.1 M PBS, with the electric potential swept from +500 to −500 mV at a scan rate of 150 mV/s [41]. A higher concentration of the target microRNA results in a more pronounced peak current in the cyclic voltammetry curve. Electrochemical measurements were performed using a potentiostat fabricated based on our previous studies [43]. All experiments were carried out at room temperature in a three-electrode system including a gold disk electrode (5 mm diameter) as a working electrode, a platinum wire as a counter electrode, and an Ag/AgCl electrode (3 M KCl solution) as a reference electrode. The electrodes were purchased from Azar Electrode (Urmia, Iran).

Although this electrochemical biosensing method provided promising results in measuring microRNA concentrations in human clinical specimens in previous studies [41], it was necessary to validate its performance in measuring microRNA concentrations in total RNA samples extracted from plants. As discussed earlier, the higher the electric peak current of the biosensor, the higher the concentration of the target microRNA in the sample is. Therefore, to quantify the output of the biosensor, a relative current response (δ*I*) was defined as the biosensor response (Equation (1))

(1) $$\delta I=\left|\frac{I_{\mathrm{microRNA}}-I_{0}}{I_{\mathrm{microRNA}}}\right|$$

where *I*_microRNA_ and *I*_0_ indicate the amount of produced current in the presence and absence of target microRNA, respectively. Standard solutions with various concentrations of a microRNA compound were used to obtain the linear range of the biosensor. The signal-to-noise method [44] was considered for determining the limit of detection (LoD) of the biosensor. Moreover, similar microRNA concentrations were analyzed by the biosensor with five replications to investigate its reproducibility.

After validating the performance of the biosensor based on acceptable linear range, LoD, and reproducibility, the concentrations of target microRNAs were measured in the total RNA samples extracted from plant shoots using the Trizol method, as outlined by Davis et al. [45].

### 2.3. Database Preparation

The data collected from the biosensor were utilized to create a matrix where each row represented a plant sample, and each column contained information about a specific nutrient or the concentration of one of the measured microRNAs. Since four levels of nutrient deficiency were studied (control, mild, moderate, and severe) for each of the four nutrient types investigated in this work (N, P, K, and S), and each experiment included five replicates, the dataset consisted of 4 × 4 × 4 × 4 × 5 = 1280 samples (or rows).

In machine learning models, not all input features contribute equally to predicting the machine output [46]. To remove the data of microRNAs that were redundant in the reliable prediction of nutrient deficiency in rice plants, the minimum redundancy maximum relevance (mRMR) feature selection algorithm was applied in this study. mRMR is a filter-based technique commonly used in machine learning and data mining to select the most informative subset of features from a larger set [47]. The goal of mRMR is to enhance model performance and reduce overfitting by selecting features that are strongly relevant to the target variable, while avoiding those that are highly correlated or redundant to each other [48]. The mRMR aims to maximize the criterion shown in Equation (2) when selecting each new feature *f*.

(2) $$\mathrm{MID}(f)=I(f;c)-\frac{1}{\left|S\right|}\sum_{s\in S}I(f;s)$$

where MID is the mutual information difference, *S* is the set of selected features, *f* is a candidate feature not yet selected, and *c* is the target variable. *I*(*f*;*c*) indicates relevance, i.e., the mutual information between feature *f* and the target *c*, and *I*(*f*;*s*) indicates redundancy, i.e., the mutual information between *f* and each already selected feature *s*.

### 2.4. Machine Learning Implementation

As shown in Figure 1, the SVM, RF, and ANN were applied to address a regression problem aimed at predicting the severity of stress. In this case, the inputs for the model were the concentrations of effective microRNAs identified by the feature selection algorithm, while the output was the level of nutrient deficiency (0, 20, 50, or 100%). Additionally, a classification problem was also considered to evaluate the models’ ability to predict the type of stress.

The radial basis function (Gaussian) kernel was used to develop the SVM model, while RF was employed with a maximum of 20 pruning levels. The ANN consisted of a three-layer perceptron network using the Levenberg–Marquardt learning algorithm, with 10 nodes (neurons) in the learning layer. Each of these models has hyperparameters, and selecting the optimal values for them can greatly enhance the model’s performance during the training process [49,50]. To optimize these hyperparameters—the kernel parameter and cost function for SVM, the minimum leaf size and parent size for RF, and the weights and biases for the hidden layer neurons in the ANN—the GA was used. Various optimization methods exist, but metaheuristic techniques, like GA, are commonly applied for this purpose [51,52]. GA is a metaheuristic method that uses a population of random chromosomes representing potential solutions to an optimization problem. Over multiple iterations (generations), chromosomes that yield better fitness values survive and are used to create offspring in subsequent generations, gradually approaching the optimal solution. In this study, the fitness function aimed to minimize the prediction error of plant stress severity using machine learning methods. The GA settings for this study included a maximum of 200 iterations (generations), a population size of 50, and a mutation rate of 2.

The MATLAB R2018b programming environment was used to implement machine learning algorithms. The five-fold cross-validation was used to train and test the machines. In this method, the database is divided into five equal parts, and in the first stage, one part of this data is selected as the test, while the other four parts are used to train the machine. In the second stage, another part is selected as the test, and the remaining data are used again for training. This operation is repeated five times so that each part is considered as the test once. The advantage of this method over other methods, such as the random selection of test data, is that all the data is examined at least once as the test. Since among the machines under evaluation, the ANN needs validation data in addition to training and test data to recognize the quality of the network training, 20% of the training data was allocated to the validation process in each stage. Accuracy (Equation (3)), precision (Equation (4)), and recall (Equation (5)) for the test data were recorded as criteria for evaluating the performance of the machines in predicting the type of stress.

(3) $$\mathrm{Accuracy}=\frac{\mathrm{TP}+\mathrm{TN}}{\mathrm{TP}+\mathrm{FP}+\mathrm{TN}+\mathrm{FN}}$$

(4) $$\mathrm{Precision}=\frac{\mathrm{TP}}{\mathrm{TP}+\mathrm{FP}}$$

(5) $$\mathrm{Recall}=\frac{\mathrm{TP}}{\mathrm{TP}+\mathrm{FN}}$$

where TP is true positives (samples for which the type of deficiency is correctly predicted), TN is true negatives (samples for which the absence of an element deficiency is correctly predicted), FP is false positives (samples for which the type of deficiency is incorrectly predicted), and FN is false negatives (samples for which the absence of an element deficiency is incorrectly predicted). The mean square error (MSE) (Equation (6)) and coefficient of determination (R^2^) (Equation (7)) for the test data were recorded as criteria for evaluating the performance of the machines in predicting stress severity levels.

(6) $$\mathrm{MSE}=\frac{1}{n}\sum_{i=1}^{n}{\left(x_{p}-x_{o}\right)}^{2}$$

(7) $$R^{2}=1-\frac{\sum_{i=1}^{n}{\left(x_{p}-x_{o}\right)}^{2}}{\sum_{i=1}^{n}{\left(x_{o}-{\bar{x}}_{o}\right)}^{2}}$$

where *x*_o_ is the stress severity level applied to the plant during the experiment, *x*_p_ is the stress level predicted by the machine, and *n* is the total number of samples. The results obtained for the five folds were averaged; so, a unique value for each evaluation criterion was reported for each machine.

In this study, Taylor’s diagram [53] was used to further evaluate the performance of each model in predicting stress severity. This diagram visually represents how well the models provide accurate predictions. In the diagram, the horizontal and vertical axes correspond to the standard deviations of the actual and predicted values, while the arcs illustrate the correlation coefficient between the predictions and the actual data. The actual data are plotted along the horizontal axis, as their correlation with themselves is 1. The arcs within the quadrant reflect the root-mean-square distance (RMSD) values.

## 3. Results and Discussion

### 3.1. Performance Characteristics of the Electrochemical Biosensor

**Figure 2:**
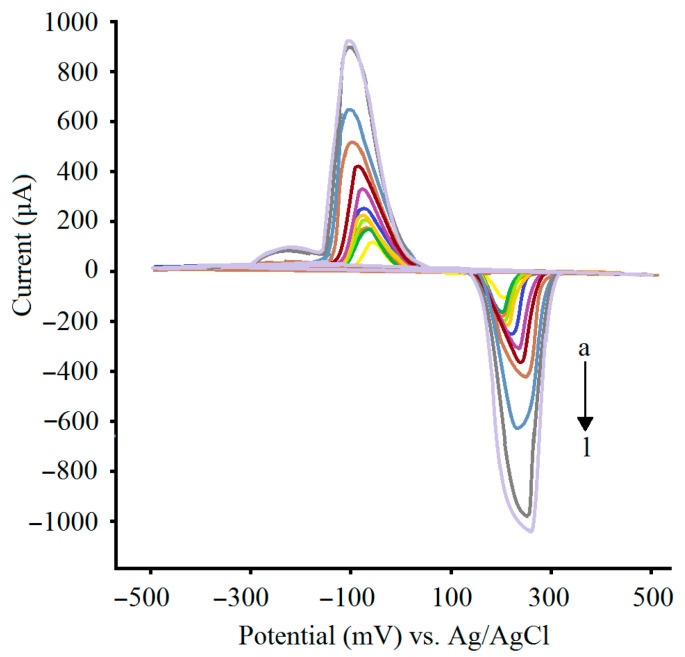
The cyclic voltammograms obtained by the biosensor at various concentrations of the studied microRNAs, (a) 0, (b) 10^−20^, (c) 10^−19^, (d) 10^−18^, (e) 10^−17^, (f) 10^−16^, (g) 10^−15^, (h) 10^−14^, (i) 10^−13^, (j) 10^−12^, (k) 10^−11^, and (l) 10^−10^ M. The cyclic voltammogram corresponding to each concentration shows a voltammogram with the closest peak to the mean peak value obtained for all studied microRNAs.

**Figure 3:**
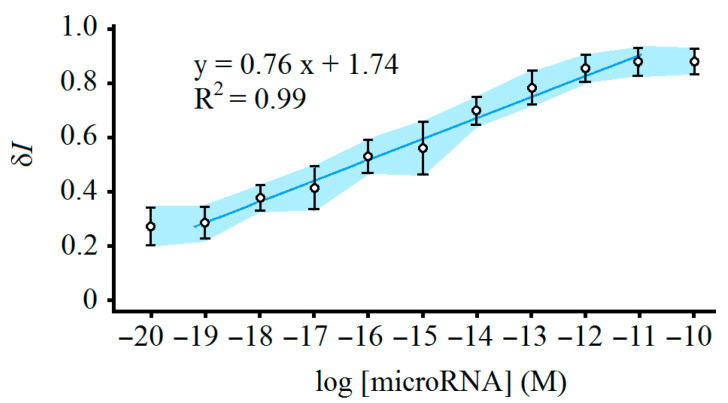
The calibration plot of the biosensor. The light blue area shows the dispersion of peak current data of studied microRNAs from the mean peak current. The darker blue line shows the linear range.

To obtain the linear range of the biosensor developed in this work, standard solutions containing a wide range of microRNA concentrations from 10^−10^ to 10^−20^ M were analyzed by the biosensor, each with five replications. Similar cyclic voltammograms were obtained for the study of 14 microRNAs. This was predictable: the sequences analyzed in this work were similar in size as they all had 21 nucleotides in their structures, and the complementary oligonucleotides were designed for full hybridization with their target microRNAs without any mismatches in their sequences (Table 1). The cyclic voltammogram corresponding with each concentration in Figure 2 shows a voltammogram with the closest peak to the mean peak value obtained for all the studied microRNAs. The calibration plot for all the studied microRNAs is shown in Figure 3. Only anodic peak currents of cyclic voltammograms were utilized in this work due to their current reproducibility [41]. The light blue area shows the dispersion of peak current data of study microRNAs from the mean peak current, indicating the acceptable reproducibility of the biosensor. According to this figure, a linear range from 10^−19^ to 10^−11^ M (the darker blue line) was considered for all the microRNAs analyzed in this work. A concentration equal to 3 × 10^−21^ M was obtained for LoD by analyzing small concentrations of the microRNAs and considering a signal-to-noise ratio of 3:1 [44]. Regarding the specificity of the biosensor, previous studies on biosensors of microRNAs involved in human cancer that work based on the absorption of positively charged PEI–Ag nanoparticles onto the negatively charged probe–target hybrid have shown that the response of such biosensors in the presence of target microRNAs is several times larger than that produced via three and more base mismatch microRNAs [41].

### 3.2. Changes in microRNA Concentration Toward Nutrient Deficiency

**Figure 4:**
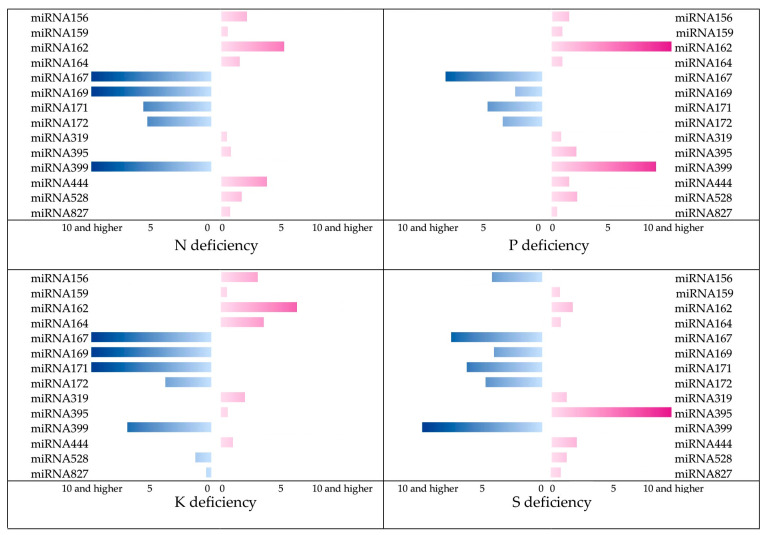
Times of changes in the studied microRNAs towards various nutrient deficiencies. Pink color shows overexpression, while the blue color shows suppression.

**Figure 5:**
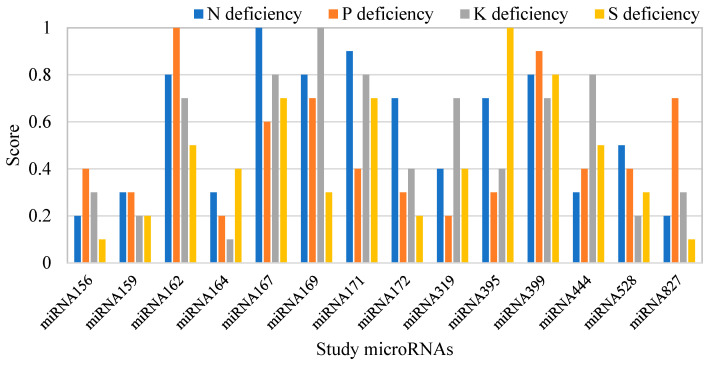
The scores of microRNAs in predicting the severity of nutrient deficiency.

After validating the performance of the electrochemical biosensor in the measurement of microRNA concentrations in treated and control plants at the femtomolar level, various microRNA sequences were measured for their possible role in the accurate prediction of plant nutrient deficiency. Figure 4 reveals the times of changes in the studied microRNAs under the severe N, P, K, and S deficiencies. Some microRNAs have changed slightly, while the concentration of some of them has either increased or decreased by more than 10 times. Figure 5 depicts the scores of the microRNAs used to predict stress levels, as determined by the mRMR feature selection algorithm. The higher the score for a model input, the more significant its impact on determining the output variable. One advantage of feature selection is that, by identifying a smaller subset of key inputs that still allow for reliable prediction performance, less important features can be excluded. This reduces both the cost of testing and the computational complexity of the system. Based on Figure 5, among the 14 microRNAs analyzed in this study, miRNA167, miRNA162, miRNA169, and miRNA395 contributed the most to predicting N, P, K, and S deficiencies in rice plants, respectively. According to Figure 4, these four microRNA compounds also show remarkable changes toward deficiencies. It is important to note that the values shown in the figure are normalized relative to the maximum value, facilitating easier comparison.

miRNA167 plays a vital regulatory role in the growth and development of rice by targeting Auxin Response Factor (ARF) genes, particularly OsARF6 and OsARF8. These ARFs are crucial components in auxin signaling pathways, which are essential for processes like root development, flower formation, and overall plant architecture. Through the regulation of ARF expression, miRNA167 helps maintain auxin homeostasis and influences organogenesis. Alterations in miRNA167 expression, either through overexpression or suppression, can result in abnormalities in reproductive organ development, decreased fertility, and changes in root morphology. Moreover, miRNA167 has been linked to stress responses, such as drought and salinity, by coordinating hormonal signaling and facilitating adaptive growth changes. The specific spatial and temporal expression patterns of miRNA167 indicate that its roles are finely tuned during various developmental stages. Therefore, miRNA167 is not only crucial for normal physiological growth but also for stress adaptation in rice, positioning it as a potential target for crop improvement strategies [54].

miRNA162 also plays a critical role in regulating gene expression, primarily by targeting DICER-LIKE1 (OsDCL1), which is a key enzyme in the biogenesis of microRNAs. By modulating OsDCL1, miRNA162 helps ensure the proper processing of other microRNAs essential for plant growth and development. This regulation is crucial for maintaining the balance and homeostasis of the small RNA pathway. Although miRNA162 is less abundant compared to other microRNAs, its influence on critical developmental processes is significant, as it indirectly affects the levels of other microRNAs that regulate various aspects of plant physiology. Disruption of miRNA162 expression can lead to altered levels of multiple microRNAs, resulting in developmental abnormalities or heightened stress sensitivity. Recent studies have also suggested that miRNA162 plays a role in stress responses, particularly in reaction to abiotic stresses such as drought and salinity. It likely influences stress-responsive gene networks, further supporting its importance in managing plant stress through its control over the miRNA pathway [55].

miRNA169 is a highly conserved microRNA in rice that plays a critical role in regulating both plant development and stress responses. Its primary targets are members of the Nuclear Factor Y subunit A (NF-YA) gene family, which act as transcription factors involved in a variety of physiological processes. In rice, miRNA169 regulates drought tolerance by modulating NF-YA expression, which in turn affects the expression of downstream genes related to stress adaptation. Under drought stress, the expression of certain members of the miRNA169 family is downregulated, leading to increased levels of NF-YA. This upregulation of NF-YA enhances the plant’s ability to tolerate drought stress. In addition to its role in stress responses, miRNA169 is also involved in nutrient homeostasis, particularly N metabolism. It plays a significant role in regulating root development, flowering, and leaf morphology. The expression of miRNA169 varies under different environmental conditions, demonstrating its central role in regulating various physiological processes in rice. Because of its broad influence on stress tolerance, nutrient regulation, and development, miRNA169 is considered a potential target for genetic engineering aimed at improving rice resilience and productivity, making it a valuable tool for crop improvement strategies [56].

miRNA395 plays a crucial role in S metabolism and stress responses in rice, primarily by regulating genes involved in sulfate uptake and assimilation, such as ATP sulfurylases (APS) and sulfate transporter (SULTR) family members. By modulating the expression of these genes, miRNA395 ensures efficient S homeostasis, which is essential for the synthesis of S-containing compounds like amino acids, proteins, and glutathione. These compounds are critical for various cellular functions, including antioxidant defense and the regulation of cellular redox balance. Under S-deficient conditions, miRNA395 expression is upregulated in rice, which enhances sulfate mobilization and redistribution throughout the plant, ensuring that essential S compounds are available for growth and development. This upregulation helps mitigate the negative effects of S limitation, enabling the plant to maintain metabolic functions under stress conditions. In addition to its role in nutrient regulation, miRNA395 is involved in the plant’s responses to abiotic stresses, such as drought and salinity. Its role in maintaining redox balance and contributing to cellular detoxification processes likely aids in enhancing stress tolerance. Altered expression of miRNA395 can lead to changes in root architecture, plant vigor, and overall stress resilience, highlighting its importance in adapting to environmental challenges. The tight regulation of miRNA395 reflects its central role in balancing plant growth and environmental adaptability. As a critical mediator of both nutrient signaling and stress resilience, miRNA395 presents a promising target for improving crop performance, particularly in the context of enhancing rice tolerance to nutrient deficiencies and abiotic stress [57].

### 3.3. Machine Performance in Predicting Stress Type and Severity Levels

Table 2 presents the performance of the machine learning algorithms in predicting both the type and severity of stress in young rice plants by having the concentrations of miRNA162, miRNA167, miRNA169, and miRNA395, identified as the four most effective features in the previous section. The table demonstrates that using microRNA concentrations as input allowed the models to effectively predict both the type and severity of mineral nutrient deficiencies in the plants. As mentioned earlier, the models were trained using both classification (to predict stress type) and regression (to predict stress severity) approaches. According to the classification results, the RF performed the best, achieving average accuracy, precision, and recall values of 0.86, 0.94, and 0.87, respectively. For all three models examined, precision values were slightly higher than the other evaluation metrics. This is because the FP rate is relatively low compared to the TP rate, as the model treats all samples with varying levels of nutrient deficiency (from mild to severe) as relevant. Consequently, the machine retrieves three times as many relevant samples as irrelevant ones, such as control samples with no deficiency. In terms of the regression approach, the RF model again showed the best performance in predicting stress severity, with average MSE and R^2^ values of 0.010 and 0.92, respectively.

Some microRNA compounds were upregulated in response to stress, while others were down-regulated (Figure 5). From a machine learning perspective, it does not matter whether microRNAs are upregulated or down-regulated. What is crucial is that the response behavior of these compounds to stress was highly specific, and the machine was able to detect this specific pattern. In some cases, when predicting different stress levels, the models occasionally predicted deficiency levels outside the expected range of 0–100%, especially in the control group, where the stress level was set to zero. Whenever the models predicted negative values or values greater than 100% for stress levels, these were adjusted to 0% and 100%, respectively, in the final machine output.

As shown in Table 2, the RF model outperformed the other two models. While ANNs are powerful tools for solving complex problems across various fields, they are often viewed as *black-box* models, meaning they lack transparency, which can hinder the ability to derive effective solutions. This lack of transparency can pose additional challenges, particularly for complex models, ultimately reducing their predictive performance [58,59]. The SVM model showed the weakest performance. Although SVMs typically perform well in medium-dimensional spaces, they struggle with complex problems involving large datasets. SVMs rely on kernel functions to map the input space to a higher-dimensional space where the data becomes more separable. However, selecting and tuning the kernel hyperparameters can be difficult and often requires extensive experimentation [60]. SVMs also assume that classes can be separated by a hyperplane, but if the classes overlap significantly, this assumption may not hold, leading to poor performance or the need for more complex methods to find an appropriate decision boundary.

If the hyperparameters of the machines used in this study were not optimized using the GA, the performance evaluation metrics would be significantly weaker, being unacceptable for accurately determining stress levels. Similar findings on the effectiveness of GA in microRNA-involved predictions are reported in our previous study in which we predicted the storage condition of strawberry fruits by having their microRNA concentrations [61]. When finding optimal values for machine hyperparameters, the GA has proven highly effective in finding optimal solutions within large and complex search spaces. In scenarios where gradient-based methods might struggle to identify the best solution, the GA provides a powerful alternative [62]. GAs work by exploring solutions through a population of particles, allowing for parallel searches across the space to quickly identify the optimal solution. Unlike traditional optimization methods, GAs are less prone to getting stuck in suboptimal solutions [63].

Table 2 also indicates that, overall, the performance of each machine was quite similar in predicting both the type and severity of nutrient deficiencies. However, the models performed slightly better when predicting the type of N deficiency in the samples, highlighting the stronger relationship between the studied microRNAs and N deficiency in rice plants. N deficiency in rice plants triggers a wide range of genes that help the plant manage limited N availability. These genes include transporters such as OsNRT2.1, OsNRT2.2, OsNAR2.1, and OsAMT1.1/1.2, which are upregulated to enhance nitrate and ammonium uptake under low N conditions. Regulatory genes like OsNLP1, OsNLP3, OsDOF18, OsGRF4, and OsTCP19 play roles in N signaling and transcriptional control of N-responsive genes. N assimilation genes, such as OsNR1/2, OsNiR, OsGS1;1/1;2, and OsGOGAT1, help convert absorbed N into amino acids and other essential compounds, with their expression adjusted according to N levels. Additionally, genes like OsDEP1 and OsCIPK23 are involved in N use efficiency and stress response mechanisms. These coordinated gene responses ensure that the plant effectively adapts to N scarcity by enhancing N acquisition, optimizing metabolism, and regulating growth to sustain development under nutrient-limited conditions [64].

The RF model also showed acceptable performance in predicting P and K deficiencies in rice. In the case of P deficiency, rice plants activate a variety of genes to boost phosphate uptake, transport, and recycling. High-affinity phosphate transporters such as OsPHT1;2, OsPHT1;3, and OsPHT1;8 are upregulated to enhance phosphate acquisition. Transcription factors like OsPHR1/PHR2 and signaling components such as OsSPX genes regulate the plant’s response to P starvation. MicroRNAs like miRNA399 and miRNA827 regulate key targets such as OsPHO2 and SPX-MFS genes. Genes involved in lipid remodeling (OsMGD2 and OsDGD1) and phosphatase activity (OsPAP10c) assist in recycling internal P, while root growth regulators like OsPTF1 and OsEXPA8 support adaptive root changes to improve P foraging. Regarding K deficiency, rice triggers genes involved in K^+^ uptake, transport, signaling, and stress adaptation. Key transporters like OsHAK1 and OsHAK5 are upregulated to enhance K absorption and distribution. Regulatory genes such as OsCIPK23 and OsCBL1 mediate K signaling. K channels like OsKAT1 and OsSKOR also respond to low K levels. Stress-responsive genes, including OsWRKYs, OsMYBs, and OsNHX1, help maintain ionic balance, while genes like OsEXPA8 and OsARF16 support root growth and structural changes to enhance K acquisition during deficiency [65].

Lastly, the machine successfully detected S deficiency. This deficiency activates genes involved in sulfate uptake, assimilation, and internal redistribution. High-affinity sulfate transporters such as OsSULTR1;1 and OsSULTR1;2 are significantly upregulated to improve sulfate absorption from the soil. Low-affinity and redistribution-related transporters like OsSULTR2;1 and OsSULTR3;5 respond to S limitation. Assimilation genes such as OsAPR1/2, OsSiR, and OsOASTL play a role in converting sulfate into essential amino acids like cysteine. Regulatory factors like OsMYB and OsbZIPs regulate sulfur-responsive pathways. Moreover, miRNA395 is strongly induced during S deficiency, targeting OsSULTR2;1 and ATP sulfurylase to maintain S homeostasis [66].

**Figure 6:**
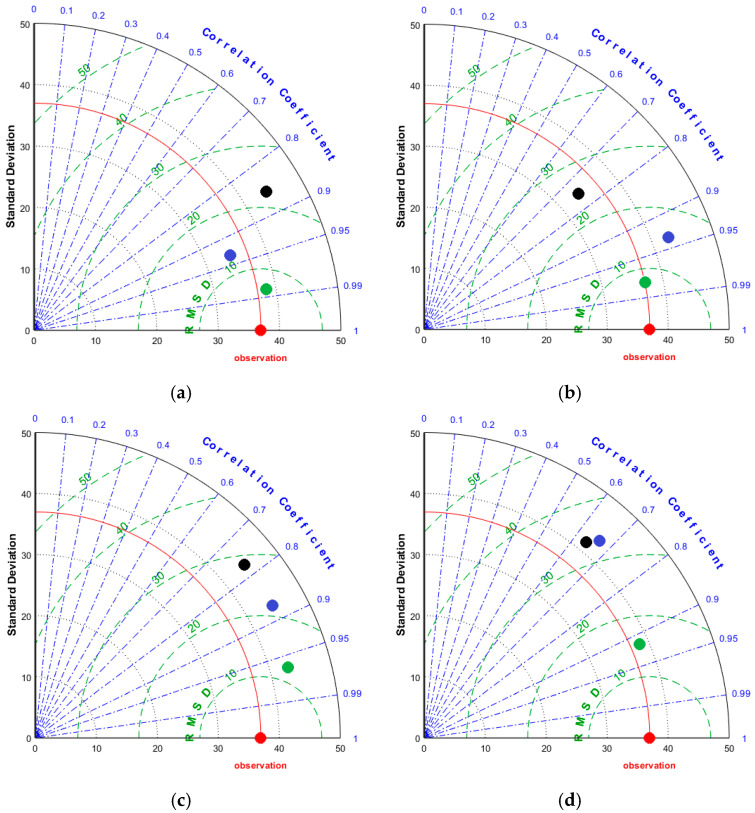
Taylor’s diagrams for the prediction of the severity of nutrient deficiency. Red-colored dots are the observed values, while the other dots belong to the predicted values using the RF (green), ANN (blue), and SVM (black). (**a**) N deficiency, (**b**) P deficiency, (**c**) K deficiency, and (**d**) S deficiency.

Figure 6 presents Taylor’s diagrams that illustrate the performance of each machine. The green points, which are closest to the actual data (represented by the red-colored observation), show that the RF model can predict the severity of nutrient deficiencies with reliable performance across all types of nutrient deficiencies. It is clear from all the plots in Figure 6 that the SVM had the greatest distance between the actual data and the predicted data, highlighting its poor performance. Furthermore, the standard deviation of the actual values of the samples and the standard deviation of the predicted values from the RF model were quite similar, indicating that the RF model is capable of accurately predicting both very high and very low output values with strong performance.

## 4. Conclusions

The findings of this study emphasize the potential role of microRNA biosensing platforms, integrated with machine learning models, for effectively predicting stress levels in plants under various nutrient deficiencies. The RF model emerged as the most promising in predicting both the type and severity of nutrient stresses. The results suggest that microRNA-based features can serve as a highly effective alternative to traditional morphological and physiological plant features, which are often time-consuming to extract and require laboratory facilities. By utilizing microRNAs, farmers, agricultural engineers, and plant scientists can gain valuable insights into plant health and stress conditions at earlier stages, potentially leading to more timely interventions and improved crop management. One of the key takeaways from this study is that not all microRNAs are equally important in predicting stress levels. Specific microRNAs were found to be highly influential in predicting certain types of nutrient deficiencies in rice, highlighting the possibility of streamlining the monitoring process. By focusing on the most relevant microRNAs, the system can provide efficient predictions without the need to measure a large number of microRNA compounds.

The proposed electrochemical biosensor has broader implications beyond rice and could be adapted for other crops as well. Moreover, it can be used to determine a wide range of environmental stressors as a portable device. To do this, the biosensor should measure the four microRNA compounds identified in this study for nutrient deficiency detection (i.e., miRNA162, miRNA167, miRNA169, and miRNA395), as well as the three microRNAs identified in our previous work for the detection of salinity, drought, and heat stresses (i.e., miRNA156, miRNA159, and miRNA393) [39]. Such biosensing platforms are the next generation of plant health monitoring systems. In addition, integrating the biosensor with machine learning algorithms implemented on portable computers offers practical and cost-effective devices. By incorporating Internet of Things (IoT) technologies, the biosensor can even transmit data wirelessly to a host computer, allowing for remote analysis and providing farmers with immediate feedback on crop health, regardless of distance.

However, there are notable challenges to the widespread adoption of this method. microRNAs are present in plants at very low concentrations, often at the femtomolar level, and their levels are influenced by environmental factors, nutrient availability, and other variables like plant diseases. This introduces a potential source of error when predicting stress under varying conditions, especially in uncontrolled agricultural environments. To address this, future research should focus on creating large, comprehensive databases that account for diverse plant conditions, growth stages, and disease scenarios. Such databases, supported by advanced machine learning models, could greatly improve the accuracy and reliability of predictions, enabling more robust applications in agriculture.

## Figures and Tables

**Table 1:** The sequences of target microRNAs and their thiolated oligonucleotides.

Target microRNA	Sequence of Target microRNA and Its Thiolated Probe
miRNA156	5′-GCUCACUCUCUAUCUGUCAGC-3′ 5′-AAAAAAAAAAGCTGACAGATAGAGAGTGAGCTTTTTTTTT-HS-3′
miRNA159	5′-AUUGGAUUGAAGGGAGCUCCG-3′ 5′-AAAAAAAAAACGGAGCTCCCTTCAATCCAATTTTTTTTTT-HS-3′
miRNA162	5′-UCGAUAAACCUCUGCAUCCAG-3′ 5′-AAAAAAAAAACTGGATGCAGAGGTTTATCGATTTTTTTTT-HS-3′
miRNA164	5′-UGGAGAAGCAGGGCACGUGCA-3′ 5′-AAAAAAAAAATGCACGTGCCCTGCTTCTCCATTTTTTTTT-HS-3′
miRNA167	5′-AGGUCAUGCUGUAGUUUCAUC-3′ 5′-AAAAAAAAAAGATGAAACTACAGCATGACCTTTTTTTTTT-HS-3′
miRNA169	5′-UAGCCAAGGAUGACUUGCCUA-3′ 5′-AAAAAAAAAATAGGCAAGTCATCCTTGGCTATTTTTTTTT-HS-3′
miRNA171	5′-UGAUUGAGCCGCGCCAAUAUC-3′ 5′-AAAAAAAAAAGATATTGGCGCGGCTCAATCATTTTTTTTT-HS-3′
miRNA172	5′-AGAAUCUUGAUGAUGCUGCAU-3′ 5′-AAAAAAAAAAATGCAGCATCATCAAGATTCTTTTTTTTTT-HS-3′
miRNA319	5′-AGCUGCCGAAUCAUCCAUUCA-3′ 5′-AAAAAAAAAATGAATGGATGATTCGGCAGCTTTTTTTTTT-HS-3′
miRNA395	5′-GUGAAGUGUUUGGGGGAACUC-3′ 5′-AAAAAAAAAAGAGTTCCCCCAAACACTTCACTTTTTTTTT-HS-3′
miRNA399	5′-UGCCAAAGGAGAAUUGCCCUG-3′ 5′-AAAAAAAAAACAGGGCAATTCTCCTTTGGCATTTTTTTTT-HS-3′
miRNA444	5′-GCUAGAGGUGGCAACUGCAUA-3′ 5′-AAAAAAAAAATATGCAGTTGCCACCTCTAGCTTTTTTTTT-HS-3′
miRNA528	5′-UGGAAGGGGCAUGCAGAGGAG-3′ 5′-AAAAAAAAAACTCCTCTGCATGCCCCTTCCATTTTTTTTT-HS-3′
miRNA827	5′-UUAGAUGACCAUCAGCAAACA-3′ 5′-AAAAAAAAAATGTTTGCTGATGGTCATCTAATTTTTTTTT-HS-3′

**Table 2:** The performance of the machines in predicting the type and severity of stress on rice plants by having microRNA concentrations.

Machine	Type of Nutrient Deficiency	Predicting the Type of Stress			Predicting the Severity of Stress	
		Accuracy	Precision	Recall	MSE	R^2^
Support vector machine	N	0.72	0.91	0.70	0.134	0.56
	P	0.64	0.87	0.61	0.095	0.63
	K	0.68	0.91	0.64	0.161	0.51
	S	0.58	0.81	0.57	0.090	0.72
	Mean	0.65	0.88	0.63	0.120	0.61
Random forest	N	0.91	0.96	0.91	0.008	0.92
	P	0.87	0.95	0.88	0.009	0.91
	K	0.86	0.92	0.89	0.011	0.93
	S	0.80	0.93	0.81	0.011	0.92
	Mean	0.86	0.94	0.87	0.010	0.92
Artificial neural network	N	0.76	0.93	0.76	0.043	0.85
	P	0.70	0.93	0.65	0.072	0.82
	K	0.77	0.91	0.78	0.035	0.89
	S	0.70	0.87	0.71	0.044	0.83
	Mean	0.73	0.91	0.72	0.049	0.848

## Data Availability

The data supporting the results of this study are available upon reasonable request from the corresponding author.
